# Implications of small-bowel transit time in the detection rate of capsule endoscopy: A multivariable multicenter study of patients with obscure gastrointestinal bleeding

**DOI:** 10.3748/wjg.v23.i4.697

**Published:** 2017-01-28

**Authors:** Carlo Maria Girelli, Marco Soncini, Emanuele Rondonotti

**Affiliations:** Carlo Maria Girelli, Gastroenterology and Digestive Endoscopy Unit, Hospital of Busto Arsizio (VA), 21052 Busto Arsizio (VA), Italy; Marco Soncini, Gastroenterology, S. Carlo Hospital, 20121 Milan, Italy; Emanuele Rondonotti, Gastroenterology, Valduce Hospital, 22100 Como, Italy

**Keywords:** Capsule endoscopy, Small-bowel transit time, Detection rate, Diagnostic yield, Small bowel, Obscure gastrointestinal bleeding, Prokinetics, Suspect small-bowel bleeding

## Abstract

**AIM:**

To define the role of small-bowel transit time in the detection rate of significant small-bowel lesions.

**METHODS:**

Small-bowel capsule endoscopy records, prospectively collected from 30 participating centers in the Lombardy Registry from October 2011 to December 2013, were included in the study if the clinical indication was obscure gastrointestinal bleeding and the capsule reached the cecum. Based on capsule findings, we created two groups: P2 (significant findings) and P0-1 (normal/negligible findings). Groups were compared for age, gender, small-bowel transit time, type of instrument, modality of capsule performance (outpatients *vs* inpatients), bowel cleanliness, and center volume.

**RESULTS:**

We retrieved and scrutinized 1,433 out of 2,295 capsule endoscopy records (62.4%) fulfilling the inclusion criteria. Patients were 67 ± 15 years old, and 815 (57%) were males. In comparison with patients in the P0-1 group, those in the P2 group (*n* = 776, 54%) were older (*P* < 0.0001), had a longer small-bowel transit time (*P* = 0.0015), and were more frequently examined in low-volume centers (*P* < 0.001). Age and small-bowel transit time were correlated (*P* < 0.001), with age as the sole independent predictor on multivariable analysis. Findings of the P2 group were artero-venous malformations (54.5%), inflammatory (23.6%) and protruding (10.4%) lesions, and luminal blood (11.5%).

**CONCLUSION:**

In this selected, prospectively collected cohort of small-bowel capsule endoscopy performed for obscure gastrointestinal bleeding, a longer small-bowel transit time was associated with a higher detection rate of significant lesions, along with age and a low center volume, with age serving as an independent predictor.

**Core tip:** There is growing evidence that a slower small-bowel transit time (SBTT) increases the diagnostic yield of small-bowel capsule endoscopy (SBCE). The present study-an analysis of a large database of consecutive, prospectively collected, complete SBCE performed for obscure gastrointestinal bleeding-confirms this finding. However, we found a correlation between SBTT and age, with age serving as an independent predictor on multivariable analysis. Prokinetics, used to increase the completion rate of SBCE, may hamper the detection rate of significant lesions and should only be used in selected patients.

## INTRODUCTION

Obscure gastrointestinal bleeding (OGB) is defined by persistent or recurrent bleeding from the gastrointestinal tract after an unremarkable esophagogastroduodenoscopy and colonoscopy. In a recent clinical guideline, the European Society of Gastrointestinal Endoscopy (ESGE) strongly recommended small-bowel capsule endoscopy (SBCE) as a first-line investigation in patients with OGB[1]. In this setting, the diagnostic yield (DY) of SBCE is highly variable, ranging from 40%-80%, depending by the clinical significance of the endoscopic finding, the degree of bowel cleanliness, and the completion rate (CR) of the small-bowel examination. Hospitalization, previous surgery or radiation, diabetes mellitus and very old age have been identified as risk factors for incomplete SBCE evaluation[2-5] that, at least theoretically, may impair the DY. To improve the CR, some clinicians administer prokinetics before capsule ingestion. However, two recent retrospective studies suggested that a prolonged small-bowel transit time (SBTT) is associated with an improved DY[6,7]. Furthermore, newer-generation devices have longer battery life, possibly minimizing the role of CR in the detection rate (DR) of significant findings.

To evaluate the role of SBTT along with other variables on the DR of significant lesions of SBCE in patients with OGB, we undertook an analysis of the Lombardy Registry, a database collecting the records of nearly all SBCEs performed for clinical purpose in a well-defined Northern Italian region.

## MATERIALS AND METHODS

### Lombardy registry

Lombardy is a highly populated region of Northern Italy, harboring approximately 10 million inhabitants. In October 2011, we implemented an SBCE database, asking every center in Lombardy performing SBCE to complete an electronic case report form (CRF) of all consecutive patients submitted to SBCE for any clinical indication. At the end of reading and video interpretation, the referring physician of the adhering center uploaded the completed CRF onto a shared Dropbox folder (Dropbox Inc. San Francisco, CA, United States). Thirty of 32 centers (see Supplementary materials) agreed to participate to data collection, which was terminated in December 2013. Centers were mostly primary or secondary care hospitals (21/30, 70%). The CRF collected demographic and clinical data, such as indication to SBCE, capsule operative system, inpatient/outpatient status, risk factors for capsule retention, previous investigations, agile patency-capsule administration, capsule retention, any complication, gastric transit time, SBTT, CR, bowel cleanliness, findings and further workup/treatment of patients with positive findings.

### Inclusion and exclusion criteria

**Figure 1:**
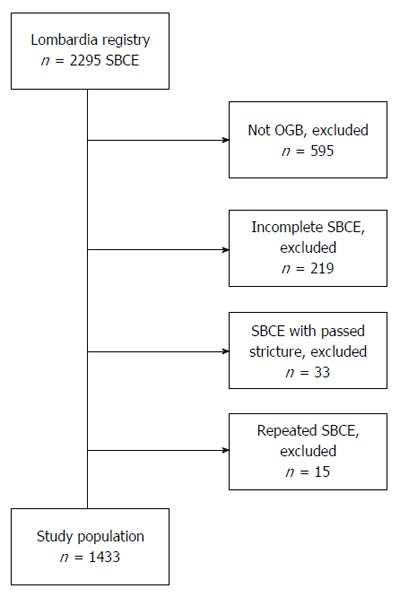
Study design and inclusion criteria. SBCE: Small-bowel capsule endoscopy; OGB: Obscure gastrointestinal bleeding.

For the aim of the present study, we included all consecutive patients submitted to SBCE for OGB of each participating center in which the capsule reached the cecum (complete small-bowel examination) without the prior administration of prokinetics. Patients with a passed small-bowel stricture, or in whom the procedure was repeated, were excluded. We adopted these selection criteria to evaluate the genuine role of SBTT on the SBCE detection rate and to avoid duplicate cases (Figure 1).

### Findings

Findings were classified using the Capsule Endoscopy Standard Terminology[8]. They were categorized in two groups, namely P0-1 and P2. P0 and P1 refer to patients with normal or negligible findings (*i.e*., lesions carrying a very low probability of bleeding), respectively, whereas P2 refers to patients with clinically significant lesions and/or luminal blood (Table 1).

**Table 1:** Classification of the findings of small-bowel capsule endoscopy

**P0-1 Group**	**P2 Group**
Normal	AVM
Lymphangectasia	Hemangioma
Small isolated phlebectasia	Mass
Lymphatic cyst	Erosion(s)
Isolated tiny red spots	Ulcer(s)
	Blood

AVM: Artero-venous malformations.

### Bowel cleanliness

Bowel cleanliness was deemed adequate by the single operator immediately after reading of the record. We adopted this subjective criterion, since - at the time of study implementation - the proposed scales of small-bowel cleansing did not undergo external validation and had poor inter- and intra-observer agreement[9].

### Center volume

The center volume was established by the number of SBCEs performed annually and was stratified into three classes: low, middle and high (≤ 20; 20-50, ≥ 50 procedures/year, respectively). These cutoff values were selected arbitrarily, in order to obtain the most balanced distribution of cases among classes.

### SBCE device and procedural protocol

Most SBCE procedures (86%) were performed by the Pillcam system (Covidien plc, Dublin, Ireland). Others (Endocapsule, Olympus Optical Co, Tokyo, Japan; Miro-Cam, IntroMedic, Seoul, Korea; OMOM capsule, Jinshan Science and Technology Group, Chongqing, China) were used on a minority of patients and were considered together in data analysis. All patients ingested the capsule in the morning in a fasting state, after a standard preparation with two liters of polyethylene glycol consumed 12-18 h before the capsule ingestion. A light snack was allowed four hours after capsule ingestion.

### SBTT

SBTT was calculated in minutes, as the time elapsed from the first frame of the duodenal bulb to the first frame of the cecum. Because of the aforementioned inclusion criteria (*i.e*., complete SBCE), gastric transit time (GTT) was not included in our data analysis. An inverse relationship between GTT and SBTT is indeed foreseeable for selection bias.

### Ethical considerations

All patients provided their written informed consent before capsule ingestion. This study was conducted in accordance with established research ethics guidelines. Permission to review patient records was granted by the Local Ethics Committee. Further specific ethical review and approval were not required because the study was considered an evaluation of previously collected SBCE records, using anonymous data previously obtained as part of routine clinical care.

### Statistical analysis

Continuous variables are presented as the mean ± SD and dichotomous variables are presented as percentages. If variable distributions were not normal (Kurtosis outside the interval between -1 and 1), variables were ranked in their interquartile ranges (IQR) and analyzed by nonparametric Mann-Whitney *U* and Spearman’s *ρ* tests. χ^2^ (2 × 2 and 2 × 3 contingency tables), Student’s *t*-test, Pearson’s R test, and multivariate stepwise regression analysis were used when appropriate. A *P* value < 0.05 was considered statistically significant. The SPSS package was used for statistical computations.

## RESULTS

**Figure 2:**
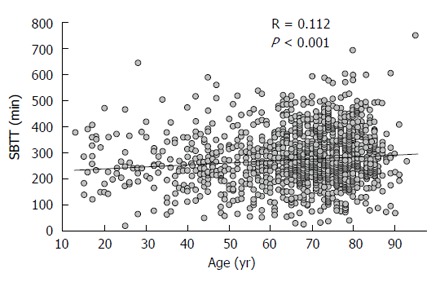
Pearson’s correlation between age (years) and small-bowel transit time (min). SBTT: Small-bowel transit time.

We retrieved and scrutinized 1433 out of 2295 SBCE records (62.4%) fulfilling the inclusion criteria (Figure 1). Patients were 67 ± 15 years of age (range: 13-95) and 815 (57%) were males. Seventy-six, 431 and 926 patients were examined in low-, mid- and high- volume centers, respectively. Small-bowel cleanliness was deemed adequate in 1376 patients (96%), and P2 lesions were encountered in 760 (54%) patients. Findings of the P2 group were artero-venous malformations (AVM) (54.5%), inflammatory lesions (23.6%), mass/tumor (10.4%), and luminal blood (11.5%). The main features of the two groups are summarized in Table 2. In comparison with patients of P0-1 Group (*n* = 657; 46%), those of P2 Group (*n* = 776, 54%) were older (69 ± 13 years *vs* 64 ± 16 years of age, *P* < 0.0001), with a longer SBTT (283 ± 105 min *vs* 269 ± 98 min, *P* = 0.0015). Furthermore, more P2 patients were examined in low-volume centers (low-volume 7% *vs* 3%; mid-volume 33% *vs* 27%; high-volume 60% *vs* 70%, *P* < 0.001), and adequate bowel cleanliness was more frequent in patients of the P2 group, with a borderline statistical significance (97% *vs* 95%, *P* = 0.06). Among variables, we found a significant correlation between age and SBTT (Pearson’s R = 0.112, *P* < 0.001; Figure 2). In the final model of multivariable analysis-including age, SBTT, and center’s volume-age was the independent predictor for the detection of P2 lesions (β = 0.16; *P* < 0.01; Table 3).

**Table 2:** Comparison between groups with significant (P2) and normal/negligible (P0-1) findings of small bowel capsule endoscopy in patients with obscure gastrointestinal bleeding

	**P2 group**	**P0-1 group**	***P* value**
*n* (%)	776 (54)	657 (46)	
Age (yr), mean ± SD	69 ± 13	64 ± 16	< 0.0001
Male gender (%)	59.0	54.5	0.09
SBTT (min), mean ± SD	283 ± 105	269 ± 98	0.0015
Pillcam SB (%)	86.3	85.3	0.61
Outpatients (%)	56.6	61	0.1
Center’s volume (%), low/mid/high	7/33/60	3/27/70	< 0.001
Adequate cleanliness (%)	97	95	0.06

SBTT: Small-bowel transit time.

**Table 3:** Product-moment correlation matrix in the final model of multiple stepwise regression

**Variables**	**Center’s volume**	**Age**	**SBTT**	**P0-1/P2 lesion**
Center’s volume	1.000	0.006	0.009	0.112
Age	0.006	1.000	0.112	0.160
SBTT	0.009	0.112	1.000	0.064
P0-1/P2 lesion	0.112	0.160	0.064	1.000

P0-1/P2 lesion is the dependent variable. SBTT: Small-bowel transit time.

## DISCUSSION

Several factors are known to influence the DY of SBCE in patients with OGB and other indications, but the role of SBTT has been only recently highlighted. In a retrospective series of 212 patients with OGB and complete small-bowel visualization, Buscaglia et al[6] showed a two-fold increase in the number of DY in patients with an SBTT longer than six hours. Westerhof and coworkers in a retrospective series of 690 consecutive patients found a correlation between SBTT and DY for all indications, but suspected Crohn’s disease[7]. One explanation of this finding may be that a slower passage of the capsule in the small-bowel may allow a better DR of significant lesions. Accordingly, the colonoscopic concept that a longer time for withdrawal of the scope corresponds to greater adenoma detection rate[10] could be directly translated to the domain of capsule endoscopy. This finding is not futile, having weight in the controversy regarding the administration of prokinetics prior to SBCE. Although prokinetics are advocated to overcome a slow gastric emptying, they, in turn can jeopardize the visualization of the entire small-bowel[11], their indiscriminate use may actually lower the DY. Metoclopramide improves the CR by reducing the gastric transit time, whereas its effect on SBTT is less clear[12]. Nevertheless, a reduction of SBTT by mosapride (a drug pharmacologically related to metoclopramide for antagonism of 5-HT3 receptors) has been reported[13]. Interestingly, Koulaouzidis and coworkers found that orally administered domperidone prior to SBCE, performed for various indications with the Pillcam system, increased the CR at expenses of a reduced DY[14]. Erythromycin, which increases the CR of SBCE without reducing the SBTT in healthy volunteers[15,16], may be the prokinetic of choice, but a controlled retrospective study was disappointing[17]. To achieve a complete examination, at least for the Pillcam SB system, the real-time display of the recorder may be helpful in selecting patients who benefit from intravenous prokinetic, by administration of i.v. prokinetic if the gastric folds are still visible after 45 min from capsule ingestion.

Our study shows that a longer SBTT increases the DR of P2 lesions in patients submitted to SBCE for OGB with complete small-bowel visualization. However, contrary to a study by Buscaglia, but in accordance with others[18], we found a correlation between age and SBTT, with age, in our investigation, being the stronger predictor of P2 findings in a multivariable analysis. One possible explanation of this discrepancy may be due to a skewness toward an aged population of our cohort and - as expected - a prevalence of AVMs in the P2 group.

Not surprisingly, we found that adequate small-bowel cleanliness was associated with an improved DR, albeit with borderline statistical significance. Conversely, the better DR of SBCE performed in low-volume centers is far from obvious. One may speculate that the limited resources constrain low-volume centers to more rigorous patient selection, or, alternatively, the reduced workload of low-volume centers allows the referring physician a longer time to review videos.

Two major limitations of this study restrict the generalizability of our findings and comparability with those of others: our inability to stratify patients into obscure-overt and obscure-occult bleeding, and our lack of data on blood loss severity. In fact, there is compelling evidence that the DY of SBCE in patients with the overt type of OGB critically depend on the time elapsed from bleeding to SBCE evaluation and by the severity of anemia[19,20]. Of course, it would be of interest examine different weights of the included variables between the two types of gastrointestinal bleeding. Furthermore, the study lacks a centralized blinded review of SBCE studies, which is a weakness for a diagnostic tool such as SBCE, which is affected by a sub-optimal inter-observer agreement[21]. Finally, we were unable to adjust our data for drugs consumption, because many drugs, especially opioids, can slow SBTT. However, this study has several strengths: its large sample size, prospective design, the participation of secondary care referral centers, and the multicenter evaluation of consecutive patients referred to SBCE, well representing real life.

In conclusion, this large multicenter prospective study of patients with OGB and complete SBCE shows that a longer SBTT increases the DR of SBCE and correlates with age, with older age serving as an independent predictor for P2 lesions. Our data argue against the customary use of prokinetics for SBCE. However, further comparative studies are needed to determine the advantage of increasing the completion rate by prokinetics at the expense of a faster SBTT.

## COMMENTS

### Background

Small-bowel capsule endoscopy (SBCE) is the first-line investigation in patients with obscure gastrointestinal bleeding (OGB). The diagnostic yield of SBCE is related to its completion rate (*i.e*., visualization of the entire small-bowel mucosa); for this reason, many clinicians use prokinetics before or during the procedure. However, small retrospective series suggested that a longer small-bowel transit time (SBTT) increases the diagnostic yield of SBCE.

### Innovations and breakthroughs

The present study - an analysis of a large database of consecutive, prospectively collected, complete SBCE performed for obscure gastrointestinal bleeding - confirms that a longer SBTT increases the detection rate of significant findings. However, the authors found a correlation between SBTT and age, with age serving as an independent predictor on multivariable analysis.

### Applications

Unselective use of prokinetics may hamper the diagnostic yield of SBCE.

### Research frontiers

Future studies are needed to show if prokinetics are useful for patients in which the gastric folds are still visible after 45 min after capsule ingestion.

### Terminology

Obscure gastrointestinal bleeding: a gastrointestinal bleeding in which the source is not detected by esophagogastroduodenoscopy and colonoscopy; it is of two types, namely: (1) obscure-overt, presenting with melena; and (2) obscure-occult, presenting with iron-deficiency anemia and positive fecal occult blood test. Prokinetic: a drug which enhances the motility of the gastrointestinal tract. Small-bowel transit time: the time elapsed from the first frame of the duodenal cap to the first frame of the cecum.

### Peer-review

This is a nice study, from a database that most will envy. Well done for this work, can be accepted following some corrections.
